# Preoperative anxiety: Is it affected by preoperative counseling and surgical technique?

**DOI:** 10.1371/journal.pone.0354701

**Published:** 2026-07-28

**Authors:** Syed F. Javaid, Hamed Aljanaahi, Saleh AlSeiari, Dana Aljaberi, Taif Aleissaee, Ahmed Mohammed, Fayez T. Hammad

**Affiliations:** 1 Department of Psychiatry, College of Medicine and Health Sciences, United Arab Emirates University, Al Ain, United Arab Emirates; 2 Tawam Hospital/STMC, Al Ain, United Arab Emirates; 3 Department of Surgery, College of Medicine and Health Sciences, United Arab Emirates University, Al Ain, United Arab Emirates; PGIMER: Post Graduate Institute of Medical Education and Research, INDIA

## Abstract

**Background:**

Preoperative anxiety (PA) is a common psychological response that can adversely affect patient satisfaction and surgical complications and outcomes. To reduce these effects, several studies were conducted to identify the factors that influence the severity of PA; however; none of the studies have examined the relationship between the PA and factors related to preoperative surgical and anesthetic counseling and the type of surgical technique.

**Methods:**

A cross-sectional questionnaire-based study was carried out between November 2023-January 2024, involving patients aged 18 years or older who were scheduled for surgical procedures at our institute. Anxiety levels were assessed using the Amsterdam Preoperative Anxiety and Information Scale (APAIS). PA was stratified into low (<11) and high (≥11) anxiety based on the total anxiety (both anesthesia- and surgery-related) scores.

**Results:**

The prevalence of clinically significant PA was 39.7%. In addition to the previously identified factors such as female gender, younger age and unemployment status, other factors which were also associated with higher PA included fewer pre-operative surgical consultations, longer duration of each surgical consultation, undergoing laparoscopic procedures instead of open surgery, referral to an internist for further evaluation of preexisting medical conditions and use of general anesthesia or uncertainty about the type of anesthesia (P < 0.05 for all). However, the number of anesthetic consultations and the surgeon’s level of experience were not significantly associated with PA. Interestingly, using illustrations and figures during consultations was also associated with higher PA (P < 0.05) while searching the internet for more information, did not have an effect.

**Conclusions:**

Preoperative anxiety remains a significant concern and was associated with factors related to gender, employment status, preoperative surgical and anesthetic counseling, surgical technique and type of anesthesia. These findings might help in formulating targeted interventions to reduce preoperative anxiety and improve surgical outcomes.

## Introduction

Anxiety is an emotional state characterized by feelings of tension, worry or situation-specific responses to perceived threats [1,2]. Surgical procedures often require significant psychological adjustment [3]. Preoperative anxiety (PA) is an emotional response related to concerns about anesthesia, uncertainty about diagnoses and prognoses, postoperative pain or fear of death [3]. For some patients, PA is among the most distressing aspect of hospital admission [4]. PA may present with restlessness and fatigue leading to a wide range of effects, including negative effects on anesthesia induction and decreased patient satisfaction with perioperative care. Post-operatively, high levels of PA have been shown to be associated with more severe pain, increased use of analgesic and anesthetic consumption, prolonged hospital stay and higher rates of complications and readmission [4–6].

In an effort to mitigate these consequences, several studies have been conducted to identify factors that influence the severity of preoperative anxiety. These studies have demonstrated that PA may affect up to half of all patients undergoing surgical procedures [3,7–9]. Various factors have been shown to influence the level of PA, including demographic characteristics such as age, gender, marital status and level of education [9]. Elements like previous personal or family history of illness and past surgical experience may also impact PA [3]. However, none of the studies have examined the relationship between PA and factors related to preoperative surgical and anesthetic counseling, including the surgeons’ level of experience, the number of surgical and anesthetist consultations, or the duration of each consultation. Similarly, aspects such as the use of illustrations and figures during explanations, the influence of internet search and the length of surgery remain under-investigated or unreported. To fill this gap in the literature, this study was performed to investigate the prevalence and determinants of PA, with particular focus on factors related to preoperative surgical and anesthetic counseling, surgical technique, and type of anesthesia.

## Materials and methods

### Study design and participants

This is an interviewer-administered questionnaire-based cross-sectional study conducted between November 2023 and January 2024, which included adult patients aged 18 years or older who were booked for surgical procedures at our institute. Patients were not eligible if they declined or were unable to provide informed consent or complete the questionnaire reliably. For the final analysis, patients with substantial missing data were excluded. No exclusions were applied based on surgical specialty, surgical technique, planned anesthetic technique or comorbidity profile. The research protocol was approved by the local Institutional Review Board (T-HREC, Ref. No. KD/AJ/995). Sample size calculation indicated that a study of 382 patients was adequate to investigate the determinants of PA in our population with a high degree of precision (95%). This was based on the results of previous studies which investigated other determinants of PA [7,8,10–15].

### Data collection

All consenting patients were interviewed in the preoperative anesthesia clinic by a trained interviewer. The questionnaire consisted of four sections: sociodemographic characteristics, surgical and anesthetic history, preoperative counseling characteristics and the Amsterdam Preoperative Anxiety and Information Scale (APAIS).

### Measures

The first section included sociodemographic information such as age, gender, employment status, educational status and residence in urban or rural areas. The second section investigated patients’ personal and family surgery- and anesthesia-related history and if they or their family members had any complications related to these procedures. The third section addressed other factors which might have also determined the level of PA including items related to the preoperative counseling process such as the number and duration of each surgical consultation, surgeon’s level of experience, the use of illustrations during counselling process, patient’s perception of the duration of surgery, number of times seen by the anesthetist, the need to be referred to an internist during the anesthesia assessment, patient’s perception of the type of anesthesia and the surgical technique (open, laparoscopic or others) and the use of internet search to search for more information. The fourth section included the Amsterdam Pre-operative Anxiety and Information Scale (APAIS).

The APAIS questionnaire has been specifically developed to assess PA in patients undergoing surgical procedures [9] and has been widely validated [16–19]. The instrument consists of six items; four anxiety-related (items 1, 2, 4, 5) and two information requirements (items 3, 6). The score of each item is graded on a 5-point Likert pattern from 1 (not at all) to 5 (extremely agree). Therefore, the total anxiety (both anesthesia- and surgery-related score) ranges from 4 to 20, while the total need for more information ranges from 2 to 10. For the anxiety score (both anesthesia- and surgery-related scores), a total of ≥11 has been recommended as the threshold for high anxiety level with clinical significance [14,20]. Therefore, in this study the patients were divided into two groups: those with low anxiety (total anxiety score <11) and those with high anxiety (total anxiety score ≥11).

### Statistical analysis

The data were analyzed using the Statistical Package for the Social Sciences (IBM SPSS Inc, Chicago, IL, version 29). In this study, comparison between different variables in the low and high anxiety groups was performed using Mann-Whitney U test for continuous variables and chi-square test or Fisher’s exact test for categorical variables. The Mann-Whitney U test was used due to the fact that the continuous variables (age, duration of surgery and duration of surgical consultations) were not normally distributed as shown by performing the Shapiro-Wilk and Kolmogorov-Smirnov tests. Each independent variable was first assessed in relation to high PA using bivariate logistic regression. Variables demonstrating statistically significant associations in bivariate analysis were considered for inclusion in the multivariate logistic regression analysis which was performed to determine the significant independent risk factors for having high level of PA. Multicollinearity has been checked using collinearity diagnostics such as the Tolerance and Variance Inflation Factor (VIF). A *P* value of <0.05 was considered statistically significant. Metric variables were expressed as mean±standard deviation (SD).

## Results

Four hundred and eleven patients were interviewed but a total of 401 were included in the final analysis. Ten patients were excluded because they had important information missing from the questionnaires or they did not meet the inclusion criteria. The mean age was 46.1 years (range: 18−98, SD: 15.8, 95% confidence interval: 44.5–47.6). Table 1 shows the scores of the Amsterdam Pre-operative Anxiety and Information Scale (APAIS) and its components.

**Table 1:** The scores of the Amsterdam Pre-operative Anxiety and Information Scale (APAIS) and its components.

	Mean (range)	SD	95% CI
**Anesthesia-related anxiety score**	4.5 (2-10)	2.8	4.3-4.8
**Surgery-related anxiety score**	5.3 (2-10)	2.9	5.0-5.5
**Sum of anesthesia and surgery-related anxiety score**	9.8 (4-20)	5.4	9.3-10.3
**Information desire score**	4.5 (2-10)	2.8	4.3-4.8
**Total APAIS score**	14.3 (6-30)	7.8	13.6-15.0

SD: standard deviation; CI: Confidence Interval.

Several factors were associated with the level of PA (Tables 2–5). Among demographic factors, young age (Table 2) female gender, higher level of education, unemployment and residency in urban areas (Table 3) were associated with higher level of PA (P < 0.05 for all).

**Table 2:** Preoperative level of anxiety score according to metric variables. Results are expressed as mean ± SD.

	Anxiety Scale		*P* Value
	Low (<11)	High (≥11)	
Age (years)	49.4 ± 16.1	40.9 ± 13.9	<0.001
Duration of Surgery (hours) as per patient	1.42 ± 1.35	2.41 ± 1.6	<0.001
Duration of each surgical Consultation (min)	20.9 ± 10.7	25.4 ± 11.3	<0.001

**Table 3:** Patients’ demographics and psychiatric history in relation to anxiety score. Results are expressed as number of patients in each group.

		Anxiety Scale		P Value
		Low	High	
Gender	Male	114	29	<0.001
	Female	127	131	
				
Education status	Illiterate	16	1	<0.001
	Primary School	12	5	
	Secondary School	33	10	
	High School	80	77	
	University	99	68	
				
Employment status	Employed	110	49	<0.001
	Housewife	54	52	
	Retired	37	15	
	Unemployed	41	43	
				
Residence	Urban	213	157	0.03
	Rural	24	7	
				
Presence of psychiatric disorder	Yes	11	6	NS
	No	231	153	

**Table 4:** Personal and family members’ surgical and anesthetic history in relation to anxiety score. Results are expressed as number of patients in each group.

		Anxiety Scale		P Value
		Low	High	
Number of previous surgical procedures	0	50	69	<0.001
	1	46	16	
	2	50	44	
	3 or more	95	31	
				
Personal history of previous surgical complications	Yes	17	21	<0.01
	No	255	108	
				
Personal history of previous anesthesia complications	Yes	14	13	<0.01
	No	262	112	
				
Family member/friend history of previous surgical/Anesthesia complications	Yes	8	39	<0.001
	No	233	121	

**Table 5:** Factors related to the pre-operative surgical and anesthetic counselling, type of surgical procedure and the pre-operative internet search in relation to anxiety score. Results are expressed as number of patients in each group.

		Anxiety Scale		P Value
		Low	High	
Number of times seen by the surgeon before procedure	1	86	65	0.008
	2	70	65	
	3	48	21	
	4 or more	35	11	
				
Number of times seen by the anesthetist before procedure	One time	195	125	NS
	Two times	48	33	
				
Referral to an internist for the assessment of pre-existing medical condition	Yes	47	77	<0.001
	No	192	85	
				
Level of performing surgeon	Consultant	207	139	NS
	Non-consultant	37	18	
				
Method of surgery	Open	112	69	<0.001
	Laparoscopic	32	72	
	Others	1	0	
	Don’t know	29	6	
				
Type of Anesthesia as per patient	General	179	101	<0.001
	Spinal	19	2	
	Others	31	13	
	Don’t know	11	45	
				
Use of Illustrations by the surgeon during counselling	Yes	96	88	0.002
	No	146	71	
				
Use of internet search by the patient	Yes	69	47	NS
	No	175	110	

Personal and family members’ surgical and anesthetic history were also associated with the level of PA (Table 4). So, higher number of previous personal surgical procedures was associated with lower levels of anxiety, whereas family member/friend history of surgical or anesthetic complications was associated with increased the level of anxiety (Table 4, P < 0.01 for all).

In the current study, factors related to preoperative surgical and anesthetic counseling, surgical technique and type of anesthesia were significantly associated with the anxiety level (Table 5). For instance, and as expected, a higher number of preoperative visits to the surgeon was associated with lower degree of anxiety whereas longer duration of each surgical consultation led to an increase in anxiety (P < 0.01 for both) (Table 5). The number of times seen by the anesthetist, however, was not significantly associated with anxiety level. On the other hand, referral to an internist before surgery for further assessment of pre-existing medical conditions was associated with higher anxiety levels (P < 0.001). The surgeon’s level of experience was not significantly associated with anxiety level. Other factors which affected the degree of anxiety were the method of surgical procedures. Laparoscopic surgery was associated with higher levels of anxiety compared to open surgery (P < 0.001) (Table 5). General anesthesia or uncertainty about the type of anesthesia was also associated with higher anxiety (P < 0.001). Unexpectedly, the use of illustrations or drawings during surgical consultations was associated with increased anxiety (P = 0.002). Surprisingly, preoperative internet searching did not affect the degree of anxiety (Table 5).

Although there were several factors which were shown to significantly determine the level of anxiety on univariate analysis, using multivariate analysis, only gender [β: 1.80, SE: 0.818, Exp(β): 6.050, P = 0.028], employment status [β: 0.418, SE: 0.272, Exp(β): 1.519, P = 0.041] and the method of surgery [β: −0.244, SE: 0.184, Exp(β): 0.783, P = 0.047] were demonstrated to be significant independent determinants of the level of the PA.

## Discussion

In the current study, we investigated, for the first time, the relationship between the level of PA and factors related to preoperative surgical and anesthetic counseling. We demonstrated that anxiety levels were lower among patients who had a higher number of surgical preoperative consultations whereas, the number of preoperative anesthetic consultations did not affect the anxiety levels. Referral to an internist during anesthetic consultation for further assessment of a preexisting medical condition was associated with higher anxiety. General anesthesia or uncertainty about the type of anesthesia were also associated with increased anxiety. Additionally, the study revealed that laparoscopic procedures, female gender and unemployment status led to higher levels of anxiety.

In this study, the association between lower levels of anxiety and a higher number of surgical consultations may reflect increased patient familiarity and trust over time which lead to emotional reassurance and sense of control during counselling [21]. However, longer duration of each consultation was associated with higher levels of anxiety. This seems counterintuitive since one might expect that longer consultations would provide more opportunities for interaction and, hence, reduce the anxiety [22]. It is difficult to ascertain from the current data the exact reason for this finding but it could be due to the fact that patients who were more anxious by nature or those who underwent more complex procedures were more likely to require longer discussions which were obviously associated with higher level of PA. Alternatively, it is possible that patients perceive longer consultations as indicative of more complex procedures with potential surgical complications; thus, shorter consultations may lead them to believe that surgery is less complex [23]. In this regard, Voogt et al indicated that overwhelming patients with excessive information might lead to miscommunication risks [24]. Regardless of the exact reason, these findings should be interpreted cautiously but should also highlight the importance of clear, structured and individualized communication during preoperative consultations [25,26].

In contrast to the surgical consultations, the number of anesthesia consultations did not affect PA levels. Although it is difficult to ascertain the exact reason for this discrepancy, this could be due to the timing of the anesthetic discussions, as they occur primarily late, leaving little time to influence patients. Furthermore, the discussions made during anesthesia visits are mostly technical and not directly linked to the surgical process. In addition, some authors have even shown that up to 25% of the patients were unaware of the fact that anesthetists are physicians which might explain the little effect on the patients’ emotional concerns [27]. Regardless of the current findings, it is intuitive to reinforce the need for a multidimensional support model, involving not only surgeons but also anesthetists, nurses, and rehabilitation teams.

Based on the findings of this study, PA was linked to patients’ views on complications. Longer procedures and the use of general anesthesia correlated with higher anxiety due to perceived risks [28–30]. Participants who were referred to an internist for a second opinion related to pre-existing medical conditions exhibited heightened anxiety probably due to interpreting the referral as indicative of greater complexity. Moreover, uncertainty about the type of anesthesia contributed to anxiety, reflecting fears of the anesthesia-related complications [20,25,31].

Another unexpected finding in the current study was the association between higher levels of PA and the use of figures and illustrations during surgical consultations. While visual tools are generally intended to improve patient understanding, they might, in some societies, trigger hidden fears about the surgical procedure and complications. This is more likely to happen in societies with limited health literacy such as our society in which a significant health literacy deficit has been previously identified [32]. This interpretation remains speculative because the present questionnaire did not ask patients specifically how they perceived the illustrations or whether these materials directly contributed to anxiety and further research is required to address this point in other societies with different levels of health literacy.

Consistent with this finding, laparoscopic surgery was also associated with higher anxiety compared with open surgery in this study. One possible explanation is that some patients may be less familiar with laparoscopic or technology-based surgical approaches, which could increase uncertainty or perceived risk [33]. In support of this, Demirel et al demonstrated that higher health literacy correlates with lower PA [34]. Similarly, Machado and colleagues noted that those with low health literacy often experience higher PA and a greater need for information [35]. Regardless of the exact reason, this remains a hypothesis, as we did not directly assess patients’ familiarity with laparoscopic surgery, their understanding of the technique, or their reasons for anxiety and it is difficult to ascertain, from this study, if the current findings are applicable to societies with high health literacy levels and further qualitative research is required to explore this point. Such insights would help to plan targeted interventions.

Another interesting finding was that internet search prior to surgery had no effect on the PA level. This could be due to the fact that patients search for information that matches their existing mood which align with their beliefs [36]. In addition, preoperative education may have satisfied some patients’ needs and expectations [37], showing no alteration to their anxiety levels.

The inverse relationship between personal surgical experience and PA has been well-documented [38,39]. Our study showed that individuals with previous history of surgical procedures had lower preoperative anxiety, likely due to a better understanding of the procedure and recovery, thus reducing fear. Additionally, participants with a personal or family history of surgical complications exhibited significantly increased PA, as awareness of complications leads to more fear and concern [8,40–42].

This study confirmed that PA was a prevalent phenomenon in our population, exhibiting comparable rates to studies conducted elsewhere [3,41,43,44]. This study also examined the effect of some of the demographic factors on the anxiety level. Younger patients showed significantly higher PA than older patients, as supported by previous research [43–45]. Older adults may possess better emotional coping strategies due to life experience, which mitigates PA [46]. Therefore, age-specific strategies, such as written information or relaxation techniques tailored for younger patients, can help reduce anxiety in this group [47].

Female participants in this study exhibited significantly higher levels of PA compared to their male counterparts aligning with the findings of Perks et al [48] and Eberhart et al [11]. Generally, women score higher on trait anxiety measures and tend to experience greater levels of emotional distress [20,49]. Their susceptibility to mental health issues associated with changes in ovarian hormones may further amplify the higher risk of developing PA [8,50].

The current study was the first to explore PA in the UAE, addressing a regional literature gap. The findings might be important for decision makers and can be used to formulate hospital policies and healthcare practices, aiding in the development of tailored pre-operative care strategies nationwide. Future work should focus on integrating pre-habilitation and structured perioperative programs that prepare patients psychologically and physically for surgery.

Despite the significant findings, this study has some limitations. Its cross-sectional design restricts the establishment of causal relationships between variables. Further, the questionnaire did not ask patients to explain why longer consultations, visual aids or laparoscopic surgery were associated with higher anxiety; therefore, the explanations offered for these findings should be considered plausible hypotheses rather than confirmed mechanisms. Additionally, reliance on self-reported data may introduce biases such as social desirability or recall bias. Although previous surgical procedures and previous personal or family surgical/anesthetic complications were assessed, we did not collect detailed information on previous cancellation or procedure rescheduling or whether the planned operation was a repeat or revisional procedure. These factors may plausibly influence PA, particularly when related to adverse prior experiences, and should be incorporated in future studies. The generalizability of our findings to other nations might be limited by differences in health literacy, healthcare access, hospital settings and patient demographics that could affect PA levels elsewhere. Furthermore, it is important to note that while some associations were statistically significant, their clinical relevance may be limited. For example, a 5-minute difference in consultation time, although statistically different, may not be practically meaningful in the patient care.

One of the other limitations in this study was the lack of reporting the correlation between the level of anxiety and level or complexity of surgery. This was due to the fact that this classification into minor, intermediate and major is still unclear and up to now there is no universal agreement on this classification [51,52]. This division does not only depend on the extensiveness of surgery but on the patient specific factors such as nutritional status and comorbidities [51,53]. So, a minor procedure in a comorbid patient should be considered a major one [51,53]. Previous studies tried to address this correlation [31,54,55], however, what would be more relevant is the patient’s perspective of the complexity of surgery, which is very difficult to ascertain and hence, this question was not asked to patients. In the current study, we have assessed parameters which reflected this complexity such as the duration of surgery and the surgical technique. Future research might be required to address this point.

In conclusion, in this study, we investigated, for the first time, the relationship between the level of PA and factors related to preoperative surgical and anesthetic counseling and surgical technique. These findings highlight the need for coordinated, targeted interventions, such as enhanced patient education and tailored support strategies, to minimize anxiety and improve surgical outcomes.

## Supporting information

S1 File Supplementary Data. (ODS)
